# The cerebral embolism evoked by intra-arterial delivery of allogeneic bone marrow mesenchymal stem cells in rats is related to cell dose and infusion velocity

**DOI:** 10.1186/scrt544

**Published:** 2015-01-27

**Authors:** Li-li Cui, Erja Kerkelä, Abdulhameed Bakreen, Franziska Nitzsche, Anna Andrzejewska, Adam Nowakowski, Miroslaw Janowski, Piotr Walczak, Johannes Boltze, Barbara Lukomska, Jukka Jolkkonen

**Affiliations:** Institute of Clinical Medicine-Neurology, University of Eastern Finland, Kuopio, 70211 Finland; Finnish Red Cross Blood Services, Helsinki, 00310 Finland; Fraunhofer Institute for Cell Therapy and Immunology and Translational Centre for Regenerative Medicine, University of Leipzig, Leipzig, 04103 Germany; NeuroRepair Department, Mossakowski Medical Research Centre, Warsaw, 02-106 Poland; Division of MR Research, Russell H Morgan Department of Radiology and Radiological Science, The Johns Hopkins University School of Medicine, Baltimore, MD 21205 USA

## Abstract

**Introduction:**

Intra-arterial cell infusion is an efficient delivery route with which to target organs such as the ischemic brain. However, adverse events including microembolisms and decreased cerebral blood flow were recently reported after intra-arterial cell delivery in rodent models, raising safety concerns. We tested the hypothesis that cell dose, infusion volume, and velocity would be related to the severity of complications after intra-arterial cell delivery.

**Methods:**

In this study, 38 rats were subjected to a sham middle cerebral artery occlusion (sham-MCAO) procedure before being infused with allogeneic bone-marrow mesenchymal stem cells at different cell doses (0 to 1.0 × 10^6^), infusion volumes (0.5 to 1.0 ml), and infusion times (3 to 6 minutes). An additional group (*n* = 4) was infused with 1.0 × 10^6^ cells labeled with iron oxide for *in vivo* tracking of cells. Cells were infused through the external carotid artery under laser Doppler flowmetry monitoring 48 hours after sham-MCAO. Magnetic resonance imaging (MRI) was performed 24 hours after cell infusion to reveal cerebral embolisms or hemorrhage. Limb placing, cylinder, and open field tests were conducted to assess sensorimotor functions before the rats were perfused for histology.

**Results:**

A cell dose-related reduction in cerebral blood flow was noted, as well as an increase in embolic events and concomitant lesion size, and sensorimotor impairment. In addition, a low infusion velocity (0.5 ml/6 minutes) was associated with high rate of complications. Lesions on MRI were confirmed with histology and corresponded to necrotic cell loss and blood-brain barrier leakage.

**Conclusions:**

Particularly cell dose but also infusion velocity contribute to complications encountered after intra-arterial cell transplantation. This should be considered before planning efficacy studies in rats and, potentially, in patients with stroke.

## Introduction

Stroke is one of the leading causes of death and chronic disability in adults in the industrialized countries. Only limited treatment options are available in the acute phase of stroke. Thrombolysis is the only established treatment, but is hampered by the narrow time window of 4.5 hours and very strict indications [1], thus leaving more than 90% of patients untreated.

Cell-based therapy is a promising experimental approach to enhance poststroke recovery. Positive treatment effects have been seen in stroke models by using different sources of cells and different delivery routes [2]. In particular, mesenchymal stem cells (MSCs) are safely and readily obtainable [3, 4]. Several delivery routes are available, but the intravenous transplantation technique is the most commonly used in both preclinical and clinical trials [5–8]. However, the current treatment strategies are far from optimal. For example, most of the infused cells are rapidly trapped in the lung, followed by their relocation to the internal organs [8, 9]. The pulmonary circulation can be circumvented by giving an intra-arterial infusion to increase the cell homing to the ischemic hemisphere, which has been claimed to enhance therapeutic outcome [10–12]. However, some adverse events related to intra-arterial cell infusion, such as micro-occlusions, have been reported [12, 13], raising safety concerns. Therefore, a careful optimization of the intra-arterial infusion procedures is needed before efficacy studies.

The complications encountered after intra-arterial stem cell transplantation seem to depend, to some extent at least, on the infusion technique, cell size, and infusion velocity [14, 15]. Janowski *et al*. [15] proposed that cell size and infusion velocity would be major determinants of micro-occlusion after intra-arterial cell injection, whereas cell dose should be adjusted when using different types of cells. In this study, we hypothesized that (a) complications of intra-arterial cell delivery would be dose dependent (that is, a lower cell dose would be safer for cell delivery); and (b) complications would be related to both infusion volume and velocity (that is, extending the infusion volume or time might mitigate some of the complications). A safety assessment was performed with laser Doppler flowmetry (LDF), magnetic resonance imaging (MRI), behavioral testing, and histology.

## Methods

### Animals

Forty-two adult male RccHan:Wistar rats (249 to 306 g; Laboratory Animal Centre, Kuopio, Finland) were maintained in a controlled environment (temperature, 20°C ± 1°C; humidity, 50% to 60%; light period, 07:00 to 19:00), with free access to food (2016S, Teklad) and water throughout the experiment. Animal care procedures were conducted according to the guidelines set by the European Community Council Directives 86/609/EEC, and this work was approved by the Animal Ethics Committee (Hämeenlinna, Finland).

### Preparation and characterization of rat bone marrow mesenchymal stem cells for transplantation

Oricell™ male Wistar rat bone marrow mesenchymal stem cells (BMMSCs; Cyagen Biosciences, Inc., Sunnyvale, CA, USA; Cat. No. RAWMX-01001, passage 2) were cultured according to the manufacturer’s instructions in OriCell MSC growth medium supplemented with 10% fetal bovine serum, 1% glutamine, and 1% penicillin-streptomycin (all reagents from Cyagen Biosciences Inc., Cat. No. GUXMX-90011). The medium was changed twice weekly, and cells were passaged when subconfluent (80% to 90% confluence). Rat BMMSCs (p5) were cryopreserved in the OriCell™ NCR cryopreservation medium (Cyagen Biosciences Inc., Cat.No. NCPF-10001). The cryopreserved cells were thawed in a water bath at 37°C before being decanted into the thawing medium containing α-MEM (Life Technologies Ltd., Cat. No. 41061-029) and 10% human serum albumin (octapharma, albunorm™). The cells were centrifuged, and medium was removed. The cells were then resuspended in phosphate-buffered saline (PBS), and trypan blue staining was used to determine cell viability and number by Countess™ automated cell counter (Invitrogen, Cat.No. C10281). For *in vivo* MRI tracking of transplanted cells, cells were incubated overnight with 25 μg/ml Molday ION Rhodamine B (BioPAL, CL-50Q02-6A-50), a superparamagnetic iron oxide formulation. The labeling efficiency was confirmed by Zeiss Axio inverted fluorescent microscope (Vert.A1).

### Sham-operation

**Figure 1:**
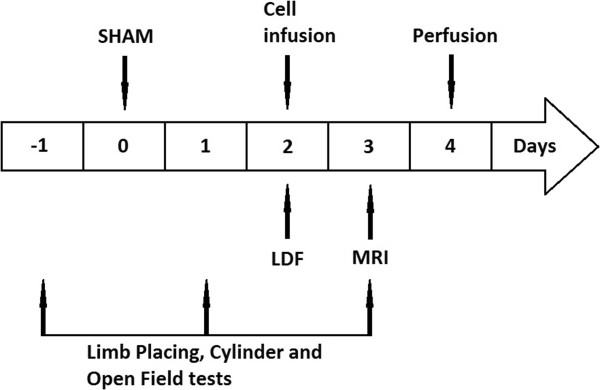
**Study design.** Cells were transplanted 48 hours after the sham-operation, and laser Doppler flowmetry (LDF) was used to monitor the cerebral blood flow during the infusion. MRI was performed on postoperative day 3, followed by the limb-placing test, the cylinder test, and the open field test. At the end, rats were perfused for histology.

The *in vivo* study design is shown in Figure 1. All rats were sham-operated to mimic the procedure of the filament-induced middle cerebral artery occlusion (MCAO). Under isoflurane anesthesia (2.0% to 2.5%) in 30% O_2_ and 70% N_2_O, the right common carotid artery (CCA), external carotid artery (ECA), and internal carotid artery (ICA) were exposed. The ECA was then cut with microscissors, and a heparinized nylon filament of 0.35 mm diameter was inserted into the stump of the ECA and advanced into the ICA. The filament was immediately retracted and the ECA was carefully closed by electrocoagulation, leaving a long ECA stump for cell infusion. Buprenorphine (0.03 mg/kg) was administered to relieve postoperative pain.

### Intra-arterial cell transplantation

Forty-eight hours after the sham-operation, rats were infused with different doses of rat BMMSCs (0.25 × 10^6^, 0.5 × 10^6^, and 1.0 × 10^6^; *n* = 6 per group) in 0.5 ml PBS during 3 minutes through the stump of the ECA with the blood flow maintained in the ICA. Control subjects (*n* = 8) were infused with 0.5 ml PBS. The infusion needle was removed after transplantation, and the ECA stump was electrocoagulated. An additional group of rats (*n* = 4) was infused with 1.0 × 10^6^ iron-labeled cells in 0.5 ml PBS within 3 minutes before MRI tracking. To explore the effects of infusion volume and velocity, 0.5 × 10^6^ cells were infused in 0.5 ml over either 3 minutes or 6 minutes, and in 1.0 ml over either 3 minutes or 6 minutes (*n* = 4 per group).

### Monitoring of cerebral blood flow with laser Doppler flowmetry

A PeriFlux System 4000 (Perimed, Sweden) with probe 407 was used to monitor the local cerebral blood flow (CBF). Before cell transplantation, an incision was made to expose the skull. The probe was placed above the sensorimotor cortex (1 mm posterior, 3 mm lateral to bregma). CBF signals were recorded starting 5 minutes before cell infusion and subsequently continuing for 30 minutes. Mean values of signal were measured before cell infusion (baseline), during cell infusion, and in 5-minute periods during 30 minutes of follow-up after cell infusion. CBF signal changes were expressed relative to baseline. The laser Doppler signal was recorded and analyzed by using PeriSoft for Windows 2.50. Area under the curve (AUC) was calculated to reveal the overall CBF changes.

### Behavioral testing

The limb-placing, cylinder, and open field tests were conducted in a blinded manner before sham-operation and 24 hours after cell transplantation to detect possible behavioral deficits related to cell infusion.

A limb-placing test was used to assess the fore- and hindlimb responses to tactile and proprioceptive stimulation [16, 17]. The test contained seven limb-placing tasks, which were scored as follows: 2 points, the rat performed normally; 1 point, the rat performed with a delay (>2 s) and/or incompletely, and 0 points, no response.

The cylinder test was used to assess possible imbalance between the impaired and the nonimpaired forelimbs [18]. The rat was placed in a transparent cylinder (Ø 20 cm) and videotaped via a mirror placed at a 45-degree angle below the cylinder. Exploratory activity was analyzed for 1 to 3 minutes. Left or right forelimb contacts or the use of both forelimbs were counted. The score for impaired forelimb was calculated as


The open field test was used to measure locomotor activity under dimmed lighting conditions to avoid the inhibitory effect of light [19]. The open field apparatus consisted of a circular arena surrounded by a 25 cm high wall. The location and movement of the experimental animal were recorded by an infrared-sensitive video camera-computer linkup. The field was divided into eight preprogrammed areas. The test lasted 10 minutes. The rat was considered moving, if the velocity was more than 1.5 cm/s. EthoVision XT 7.0 (Noldus, The Netherlands) was used to record and analyze the data.

### Magnetic resonance imaging

MRI was performed 24 hours after cell transplantation by using a Bruker 9.4 T horizontal scanner to detect micro-occlusions or hemorrhages. The rats were anesthetized with 2% to 2.5% isoflurane (5% for induction) in a gas mixture of 30% O_2_/70% N_2,_ delivered via a nose mask. A RARE sequence with the following parameters was used to acquire T_2_-weighted images: repetition time (TR) = 3.0 s, echo time (TE) = 32 ms, average = 16, matrix size of 256 × 128, field-of-view (FOV) = 30 mm × 30 mm; 15 slices with 1-mm slice thickness. T_2*_-weighted multislice images were acquired with the following parameters: TR = 750 ms, TE = 3.0 ms, TE2 = 6.0 ms, average = 6, FOV = 25.6 mm × 25.6 mm, 15 slices with 1-mm slice thickness. Lesion volumes were analyzed with MATLAB R2012a, Aedes 1.0. MRI images were also scored by using the following semiquantitative scaling: 0 point, no lesions; 1 point, one to five focal lesions (≤2 mm); 2 points, five to 10 focal lesions; 3 points, more than 10 focal lesions or confluent lesions.

### Histopathology

One day after MRI, the rats were perfused with 0.9% NaCl followed by 4% paraformaldehyde in 0.1 *M* phosphate buffer (PB) (pH 7.4). The brains were carefully removed and postfixed in 4% paraformaldehyde overnight, and then kept in 30% sucrose in 0.1 *M* PB for 5 days. Brain sections (35 μm) were cut by using a sliding microtome, with the sections being stored in antifreeze solution at -20°C.

Nissl staining and modified Gallyas silver staining [20] were used to detect lesions and to visualize degenerating terminals and cell bodies in sections matched with MRI images.

IgG staining was used to assess the blood-brain barrier leakage [21]. The sections were washed in PB 3 times, incubated in 1% H_2_O_2_ for 15 minutes, blocked in 2% normal goat serum for 2 hours, followed by incubation with biotinylated sheep anti-rat IgG (1:200; AbD Serotec, Oxford, UK) for 48 hours at 4°C. The sections were then rinsed with Tris-buffered saline with 0.5% Triton X-100 and incubated with streptavidin-horseradish peroxidase conjugate (1:1,000; GE Healthcare, UK) for 1 hour, and developed with diaminobenzidine for 4 to 6 minutes.

Prussian blue staining was used to visualize the iron-labeled BMMSCs in the brain [22]. The sections were incubated with 2% potassium ferrocyanide in 2% HCl for 5 minutes and counterstained with neutral red.

All images were captured by using a Zeiss Axio Imager M2 microscope (Carl Zeiss GmbH, Germany), with an AxioCam ER camera.

### Statistical analysis

The analyses were performed with SPSS software (version 19). CBF changes, lesion size, MRI scores, and results from behavioral tests were compared by using a Kruskal-Wallis test followed by a Mann-Whitney test. The Spearman rank correlation coefficient was used to evaluate correlations between lesion size, behavioral results, and cell dose. The level of significance was *P* <0.05. Data are presented as mean ± standard deviation (SD).

## Results

### LDF monitoring of cell transplantation

**Figure 2:**
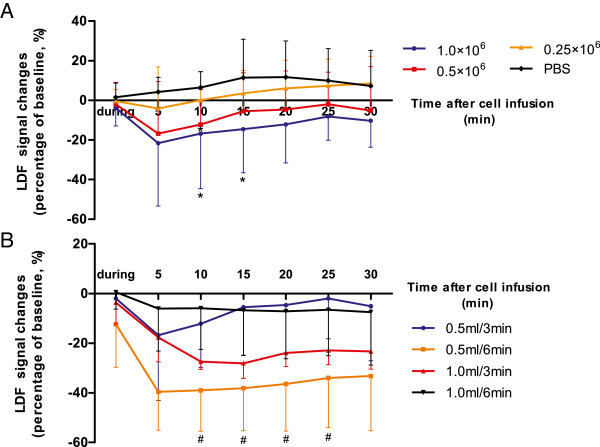
**Changes of cerebral blood flow (CBF) monitored by laser Doppler flowmetry (LDF). (A)** Changes in CBF in rats infused with different doses of cells. CBF is reduced as the cell dose is increased. *P <0.05 compared to PBS group. **(B)** Changes in CBF in rats infused with different volumes and in different time spans. ^#^ *P* <0.05 compared with the 0.5 ml/3 minute group.

A cell dose-related reduction occurred in the CBF in rats after cell transplantation. In contrast, CBF seemed to increase slightly in the control group (Figure 2A). In the 0.25 × 10^6^ group, a slight decline was noted in the CBF at the beginning, but the flow did return to a level similar to that in control group by the end of the 30-minute follow-up. Instead, CBF remained reduced in the groups receiving the two higher cell doses in comparison to the control group throughout the follow-up. The AUC value of the 1.0 × 10^6^ group (-407.02 ± 566.58) was significantly lower than that in controls (243.46 ± 378.07; *P* <0.05). The AUC value correlated negatively with the cell dose (r = -0.48; *P* <0.05). When different infusion volumes and velocities were tested, CBF decreased extensively in 0.5 ml/6-minute group (Figure 2B). The AUC value in the 0.5 ml/6-minute group (-1,069.01 ± 547.21) was significantly lower than that in the 0.5 ml/3-minute group (-225.99 ± 495.80; *P* <0.05). No significant difference was found between the other groups.

### MRI monitoring of embolisms and hemorrhage after cell transplantation

**Figure 3:**
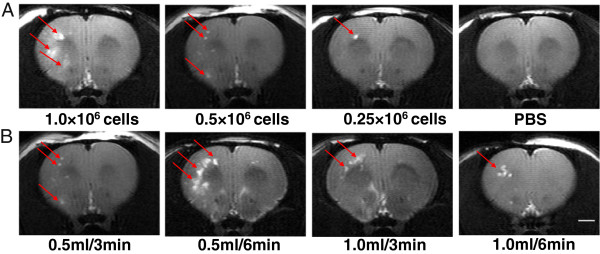
**MRI images. (A)** Typical microembolisms in rats receiving different cell doses. **(B)** Typical micro-occlusions in groups with different infusion volumes delivered over different time spans. Scale bar, 2 mm.

In all of the cell infused groups, ischemic lesions were detected. They were located mainly in the cortex and subcortical white matter (Figure 3), but some were also detected in the striatum and even the brain stem. No signs of hemorrhage were observed. In rats receiving PBS or 0.25 × 10^6^ cells, only one microembolism in one rat of each group was observed in the white matter. The number of embolisms increased with cell dose, and in some cases, they even fused together or appeared on the contralateral hemisphere. A cell dose-related increase in lesion size was found (r = 0.807; *P* <0.001) and MRI score (r = 0.846; *P* <0.001) (Table 1). No significant difference was found between the groups with different infusion volumes and velocities, although the MRI score was the highest, and lesion size was the greatest in the 0.5 ml/6 minute group (Table 2).

**Table 1:** **MRI results in rats receiving different cell doses**

	PBS	0.25 × 10 ^6^	0.5 × 10 ^6^	1.0 × 10 ^6^
Rats with micro-occlusions/total	1/8	1/6	5/6	5/6
MRI score	0.13 ± 0.35	0.17 ± 0.41	1.67 ± 0.82**^##^	2.67 ± 0.82**^##^
Lesion size (mm^3^)	0.31 ± 0.88	0.94 ± 1.99	35.87 ± 21.86**^##^	194.36 ± 165.94**^##^

**P <0.01 compared with PBS group; ^##^ *P* <0.01 compared with 0.25 × 10^6^ group.

**Table 2:** **MRI results in rats receiving 0.5 × 10** ^**6**^ **cells with different infusion volumes in different time spans**

	0.5 ml/3 min	0.5 ml/6 min	1.0 ml/3 min	1.0 ml/6 min
Rats with micro-occlusions	5/6	4/4	2/4	3/4
MRI score	1.67 ± 0.82	3.00 ± 0.00	1.50 ± 1.29	2.50 ± 1.00
Lesion size (mm^3^)	35.87 ± 21.86	197.83 ± 184.30	41.78 ± 38.87	150.35 ± 236.69

### Behavioral results after cell transplantation

**Figure 4:**
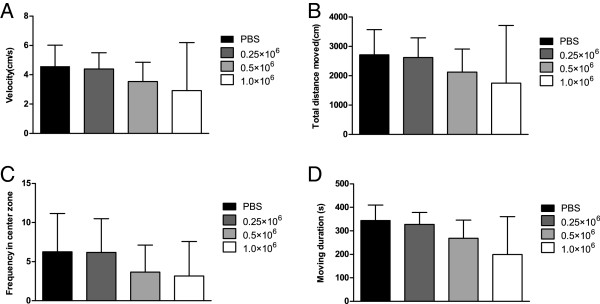
**Open field test in rats infused with different cell doses.** Velocity **(A)**, total distance moved **(B)**, frequency in center zone **(C)**, and moving duration **(D)** 24 hour after cell infusion were chosen to assess general motor function. Although no significant difference was found between the groups, the motor function after cell transplantation seemed to decrease as the cell dose increased, which was confirmed with Spearman correlation analyses.

In groups with different cell doses, the mean limb-placing score for left fore/hindlimbs in 1.0 × 10^6^ group (11.17 ± 5.04) was significantly lower than the mean score (14) in the other groups (*P* <0.01). No significant difference was found between groups in the cylinder test. With respect to the open field test, no significant difference was observed between groups after cell transplantation, but velocity, total distance moved, and moving duration all negatively correlated with cell dose (r = -0.423; r = -0.423; r = -0.494, respectively; *P* <0.05) (Figure 4).

In groups with different infusion volumes or velocities, the mean limb-placing score of left limbs (10.75 ± 3.95) in 0.5 ml/6-minute group was significantly lower than the mean score (14) in both 0.5 ml/3-minute and 1.0 ml/3-minute group (*P* <0.05), but not in 1.0 ml/6-minute group (11.25 ± 5.50). No significant differences in the performance in the cylinder or open field tests were found between groups.

### Histologic changes after cell transplantation

**Figure 5:**
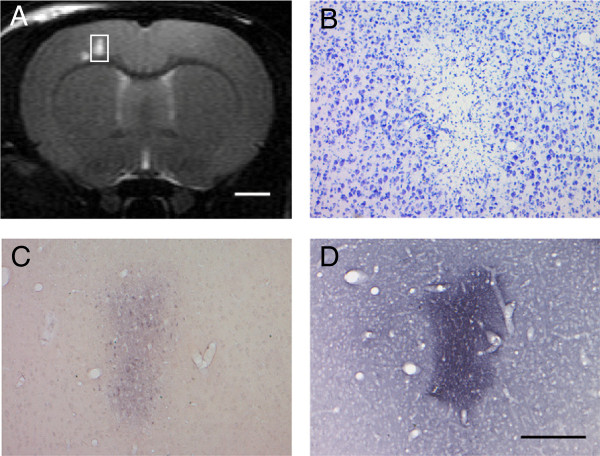
**Histology after intra-arterial cell infusion in rats. (A)** T_2_-weighted coronal slices from a rat infused with 0.5 × 10^6^ cells in 0.5 ml within 3 minutes. The white boxed region reveals a typical microembolism. Nissl staining **(B)**, silver staining **(C)**, and IgG staining **(D)** show the pathologic changes in the boxed region. Scale bar in **(A)** is 2 mm, and for **(B)-(D)** is 250 μm.

Nissl and silver staining revealed focal ischemic damage in the same region in which the lesion was detected with MRI (Figure 5A). Signs of severe degenerative process were present 2 days after intra-arterial cell delivery (Figure 5B,C). IgG staining detected a leakage of the blood-brain barrier in the lesion core (Figure 5D).

### Tracking of iron-labeled cells

**Figure 6:**
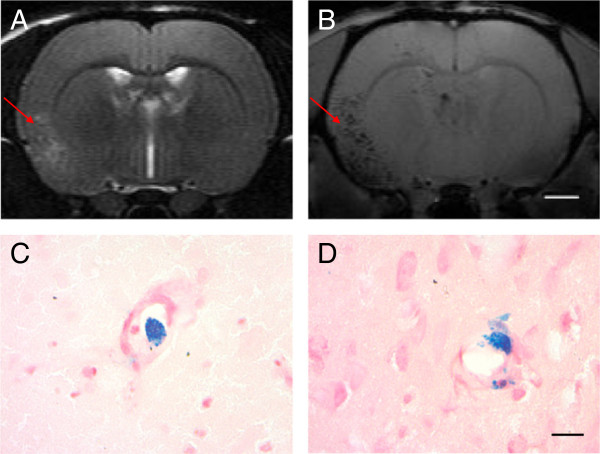
**Tracking of infused cells. (A)** T_2_-weighted image of a rat infused with 1.0 × 10^6^ iron-labeled cells in 0.5 ml within 3 minutes. **(B)** T_2*_ image at the same level. Cell infusion evokes hypointense spots on T_2*_ sequence primarily located in the ipsilateral hemisphere within and around the lesions seen in T_2_. Prussian blue staining reveals cells inside the microvessel and attached to the vascular wall **(C)**. It is also evidence that cells had migrated across the vascular wall and disaggregated **(D)**. Scale bar for **(A, B)** is 2 mm, and for **(C, D)** is 20 μm.

The extent of intravascular cell entrapment 24 hours after cell infusion (T_2*_ images) was associated with lesion number and size (T_2_ images). The T_2*_ signal was found primarily in the ipsilateral hemisphere, and it was located mainly in close proximity to the lesions (Figure 6A,B). The T_2*_ signal was rarely seen in rats without embolisms. Prussian blue staining (Figure 6C,D) revealed results consistent with the T_2*_ images, that is, cells being trapped in the microvessels, but no major aggregates were found.

## Discussion

Intra-arterial cell delivery has been postulated to enhance the homing efficiency to the target organ, which may be associated with additional therapeutic benefits, particularly in the brain. However, adverse events after intra-arterial cell delivery, such as micro-occlusions and compromised CBF, were recently reported in rodent models, raising safety concerns. Cell size, infusion velocity, and technique seem to be important determinants of safety [14, 15, 23]. Our study reveals the relation particularly between cell dose and the severity of complications, as detected by LDF, MRI, behavioral testing, and histology.

### Lower cell dose is safer for intra-arterial delivery of BMMSCs

In our study, we used ECA for cell transplantation with preserved blood flow in the ICA. Previously, an infusion velocity of 0.2 ml/min or lower has been postulated to be safe for rats [15]; thus we applied the rate of 0.5 ml/3 minutes to test different cell doses. When PBS was infused at a rate of 0.5 ml/3 minutes, the average LDF signal did not change or slightly increased, probably as a result of hemodynamic changes after the injection of vehicle into the cerebral artery. In contrast, an increasing cell dose evoked a reduction in CBF, which resulted in an increase in the numbers of ischemic lesions, as detected by MRI and sensorimotor function impairment. Therefore, lower cell doses were associated with fewer adverse events. Both MRI, 24 hours, and histology, 48 hours, after infusion revealed microembolisms as being the most likely explanation for the observed CBF decline.

Thrombi formed at damaged endothelium after filament retraction may have caused the minor downstream ischemic lesions occasionally observed in the control group. This would be a technical complication in the filament model that should be taken into account. The size of BMMSCs is relatively large (diameter approximately 20 μm), and thus the cells may be trapped in the cerebral microvessels and cause focal ischemic lesions. Despite attempts to avoid cell aggregation by suspending the cells rigorously before infusion, it is not possible to be sure that single-cell aggregates are not formed before or during the infusion. Those aggregates may also have caused microemboli in some individual cases.

In both scenarios, reducing the cell dose seems to be one way to prevent such events. In the present study, we found a dose of 0.25 × 10^6^ to be safe (that is, the complication rate was similar to that found with controls). Filtering cells before infusion might prevent aggregations, but might also result in a loss of cells. Three-dimensional spheroid cultured MSCs or flow cytometry-based pulse-width assay can also be considered as techniques to reduce or detect cell aggregates in the future [24, 25].

### Infusion velocity is closely related to the safety of intra-arterial cell delivery

Our data suggest that increasing the infusion volume (1.0 ml/3 minutes versus 0.5 ml/3 minutes) in an attempt to reduce the cell concentration, did not reduce CBF drop or lesion size. One may speculate that a rapid infusion could affect the hemodynamics, and then trigger endothelial injury, microthrombosis, or forced opening of the blood-brain barrier. Hence, we prolonged the infusion time in an attempt to prevent such adverse events. However, extending the infusion time (0.5 ml/6 minutes versus 0.5 ml/3 minutes) worsened CBF decrease. This may be explained by the higher probability of cell aggregates forming during the prolonged infusion times, which may ultimately result in even greater complications than with rapid infusion.

In line with the LDF results, the MRI score and lesion size seemed to increase in the 0.5 ml/6 minute group, although no significant difference was found, which might be due to the rather small number of animals per group and the extensive variations. Hence, a slower infusion may not be necessarily beneficial despite preventing the hemodynamic complications encountered with high injection velocities. When we extended both infusion volume and infusion time (1.0 ml/6 minutes), CBF was similar to that seen in the 0.5 ml/3 minute group (same net infusion velocity), although with a higher MRI score and a larger lesion, which might be due to the greater variation (Table 2). Thus, infusion velocity, rather than simply infusion volume or infusion time, should be considered to be more responsible for the development of microembolisms after intra-arterial MSC delivery.

### Open field test is sensitive at detecting randomly distributed microembolisms

In the present study, microembolisms were found mainly within the ICA territory, but some were also observed in the posterior circulation (brain stem) and occasionally even in the contralateral hemisphere. Given the random distribution of microembolisms and location in the white matter, it was a challenge to find a sensitive behavioral test that could assess possible functional consequences. This was also reflected as an apparent discrepancy in the occurrence of complications in 1.0 × 10^6^ and 0.5 × 10^6^ groups (that is, some of the rats were not found to have any apparent impairment in the limb-placing test or in the cylinder test, despite the presence of MRI lesions). With respect to the open field test, although no significant group difference was found in the rats infused with different doses of cells, which might be due to the high variation between animals, a significant correlation appeared between cell dose and locomotor activity. Therefore, the open field test appears to be the most sensitive technique for detecting a minor, cell dose-related decrease in motor activity [19, 26].

### Rapid disappearance of allogeneic BMMSCs after intra-arterial transplantation

Only limited information is available about the intra-arterial delivery of allogeneic cells [9, 14, 27, 28]. Recently Khabbal [27] reported different clearance rates of rat and human BMMSCs from the brain after intra-arterial infusion, with human BMMSCs disappearing more rapidly than rat cells. However, it is unclear whether the differential cell behavior has any functional impact. Moreover, despite initial effective homing to the brain, both rat and human cells disappeared almost completely within 1 or 2 days [27, 29]. Similarly, the T_2*_ signal extinctions were observed in the brain with MRI at 24 hours after infusion in the present study, but histology at 48 hours after infusion revealed fewer cells in the brain. More important, the brief presence of MSCs was associated with permanent damage with high cell doses.

Thus, one major task for future studies will be to reduce the effective therapeutic dose and to improve engraftment, which should be reflected in functional improvements.

### Limitations of the present study

The present study has several limitations. First, only sham-operated rats were used because the microembolisms caused by MSC infusion might be masked by the large corticostriatal lesions typical of most MCAO models. In addition, one cannot exclude the possibility that cells behave differently under postischemic circumstances. Another limitation is that we used the preoperated ECA for cell infusion. It might be possible that reopening the artery releases some blood emboli, causing technical micro-occlusion such as that occasionally observed in the control group. Moreover, because of the random location of lesions, LDF might not be sensitive enough to detect CBF changes in deep white matter and brain structures out of the ICA territory. For the same reason, it was challenging to detect sensorimotor impairment.

Finally, the follow-up period was limited to 3 days (that is, no assessment was made of the long-term outcome). As the observed functional deficits may be transient or minor with respect to the potential therapeutic improvements achieved by MSC, long-term efficacy studies with a safe dose of rat BMMSCs are warranted in stroke rats.

## Conclusions

The safety of allogeneic intra-arterial BMMSC transplantation is dose dependent. Infusion velocity is also closely related with safe intra-arterial administration. Therefore, cell dose and infusion velocity should be carefully optimized, according to which cell types are being delivered, as well as to the study design when planning future preclinical and clinical efficacy studies.

## Abbreviations

AUC: Area under curve
BMMSCs: bone marrow mesenchymal stem cells
CBF: cerebral blood flow
CCA: common carotid artery
ECA: external carotid artery
FOV: field of view
ICA: internal carotid artery
LDF: laser Doppler flowmetry
MCAO: middle cerebral artery occlusion
MRI: magnetic resonance imaging
MSC: mesenchymal stem cell
PB: phosphate buffer
PBS: phosphate-buffered saline
SD: standard deviation
TE: echo time
TR: repetition time.
